# Automatic and label-free detection of meningioma in dura mater using the combination of multiphoton microscopy and image analysis

**DOI:** 10.1117/1.NPh.10.3.035006

**Published:** 2023-07-10

**Authors:** Na Fang, Zanyi Wu, Rong Chen, Zhongjiang Chen, Limei Zheng, Tingqi Yang, Ling Wang, Xingfu Wang, Dezhi Kang, Jianxin Chen

**Affiliations:** a Fujian Medical University, Department of Ophthalmology and Optometry, Fuzhou, China; b The First Affiliated Hospital of Fujian Medical University, Department of Neurosurgery, Fuzhou, China; c The First Affiliated Hospital of Fujian Medical University, Department of Pathology, Fuzhou, China; d Fujian Normal University, China Key Laboratory of OptoElectronic Science and Technology for Medicine of Ministry of Education, Fujian Provincial Key Laboratory of Photonics Technology, Fuzhou, China

**Keywords:** multiphoton microscopy, two-photon excitation fluorescence, second-harmonic generation, dura mater, meningioma

## Abstract

**Significance:**

To prevent meningioma recurrence, it is necessary to detect and remove all corresponding tumors intraoperatively, including those in the adjacent dura mater.

**Aim:**

Currently, the removal of meningiomas from the dura mater depends solely on cautious visual identification of lesions by a neurosurgeon. Inspired by the requirements for resection, we propose multiphoton microscopy (MPM) based on two-photon-excited fluorescence and second-harmonic generation as a histopathological diagnostic paradigm to assist neurosurgeons in achieving precise and complete resection.

**Approach:**

Seven fresh normal human dura mater samples and 10 meningioma-infiltrated dura mater samples, collected from 10 patients with meningioma, were acquired for this study. First, multi-channel mode and lambda mode detection were utilized in the MPM to characterize the architectural and spectral features of normal and meningioma-infiltrated dura mater, respectively. Three imaging algorithms were then employed to quantify the architectural differences between the normal and meningioma-infiltrated dura mater through calculations of the collagen content, orientation, and alignment. Finally, MPM was combined with another custom-developed imaging algorithm to locate the meningioma within the dura mater and further delineate the tumor boundary.

**Results:**

MPM not only detected meningioma cells in the dura mater but also revealed the morphological and spectral differences between normal and meningioma-infiltrated dura mater, providing quantitative information. Furthermore, combined with a self-developed image-processing algorithm, the precise borders of meningiomas in the dura mater could be accurately delineated.

**Conclusions:**

MPM can automatically detect meningiomas in the dura mater label-free. With the development of advanced multiphoton endoscopy, MPM combined with image analysis can provide decision-making support for histopathological diagnosis, as well as offer neurosurgeons more precise intraoperative resection guidance for meningiomas.

## 1 Introduction

**Fig. 1:**
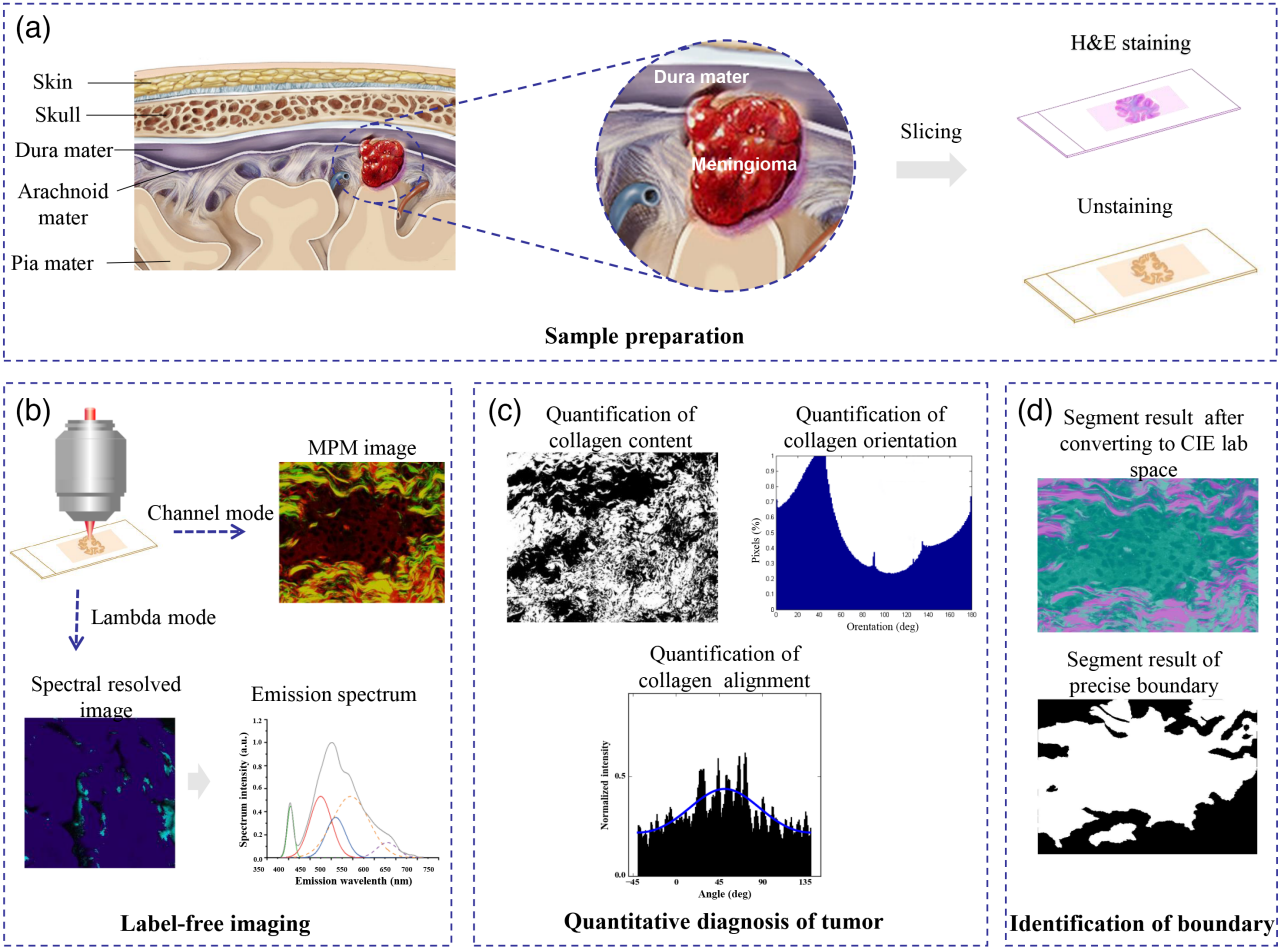
Schematics of MPM and identification steps of meningiomas in dura mater. (a) Flow of sample processing. (b) Architectural and spectral features of dura mater captured using MPM based on the multiphoton imaging and multiphoton spectrum. (c) Quantitative diagnostic information provided from MPM combined with the image processing algorithm. (d) Precise borders of meningiomas in dura mater rapidly delineated via MPM combined with the image processing algorithm. This can offer neurosurgeons more precise intraoperative resection guidance for meningiomas.

Meningiomas are the most common meningeal tumors, accounting for more than 30% of primary brain tumors.^1^ In general, meningiomas are benign (noncancerous) and widely attached to the dura, and they grow slowly without infiltrating the adjacent dura mater.^2^ However, they can cause severe morbidity. Although resection is recognized as the primary treatment for patients with meningiomas, more than 30% of patients who undergo resection experience recurrence and require another surgery within 15 years.^3^ Factors contributing to meningioma recurrence, including patient sex, age, mitotic index, extent of surgical resection, and histological subtype, have been discussed in previous studies.^4^^,^^5^ Simpson et al. proposed that a residual meningioma in the adjacent dura mater [Fig. 1(a)] is one of the most significant factors causing meningioma recurrence.^5^ This hypothesis was proven in later studies.^2^^,^^6^ Therefore, it is important to surgically detect and remove all meningioma nests from the adjacent dura mater.

Magnetic resonance imaging (MRI) is now regarded as the most specific and sensitive imaging modality for the preoperative diagnosis and grading of meningiomas, providing essential information about meningiomas, such as tumor size, location, consistency, and adjacent soft tissue structures.^7^^–^^9^ Nevertheless, limited by the spatial resolution of MRI, intraoperative meningioma detection is still dependent on a histopathologic diagnosis to reveal the microstructures of the lesion and adjacent dura mater at the cellular level. However, current clinical histopathological techniques remain constrained by multiple workflows, such as complicated protocols and exogenous markers. In addition, with the increased demand for biopsies of various diseases and the current shortage of pathologists, traditional diagnostic pathology methods further increase the burden and responsibilities of pathologists.

In meningioma surgery, in addition to detecting the meningioma, it is important to determine the surgical scale. The Simpson grading system, proposed in 1957 by Donald Simpson in his article “The recurrence of intracranial meningiomas after surgical treatment,” has been the gold standard for defining the surgical scale of meningioma surgery. Simpson categorized the scale of excision into five grades: grade I, macroscopic complete tumor resection with removal of the affected dura and bone; grade II, macroscopic complete tumor resection with coagulation of the affected dura; grade III, macroscopic complete tumor resection; grade IV, subtotal tumor resection; and grade V, decompression with or without biopsy. This scale has played an important role in the surgical resection of meningiomas for more than 60 years. However, the Simpson’s scale has some limitations. First, this system is currently only applicable to WHO grade I meningiomas and is not applicable to surgery for recurrent meningiomas or other WHO-grade meningiomas. Second, this system is sometimes subjective and inaccurate because it is based on an intraoperative visual assessment of the resection. Third, the infiltration and proliferation potential of the dural tail may vary widely based on the tumor location, as does the molecular biology of the tumor, rendering this universal scale for meningiomas in all locations unfeasible. Therefore, there is an urgent need to develop a new technique that can more precisely and objectively determine the scale of resection and lesion boundaries.

Several alternative optical-imaging techniques have been proposed to overcome these limitations. UV surface excitation microscopy^10^ and light-sheet microscopy^11^ can provide high-resolution images at high speeds; however, neither technique is label-free. Optical coherence tomography^12^ and photoacoustic microscopy^13^ enable the *in-vivo* label-free imaging of intact tissues at higher penetration depths without tissue processing. Computational diffraction tomography has the advantage of allowing for the observation of cellular sub-structures.^14^ Inevitably, these techniques are usually combined with physical model algorithms that may pose challenges for medical researchers.

Multiphoton microscopy (MPM), which is based on two-photon-excited fluorescence (TPEF) and second-harmonic generation (SHG), has evolved into a label-free, reliable, and sensitive optical technology for clinical research.^15^^–^^19^ TPEF is an absorptive process in which a fluorophore simultaneously absorbs two photons with lower energy and emits a single fluorescent photon with higher energy. SHG is a coherent scattering process in which two photons with lower energies are combined to create a single photon with exactly twice the energy. Many intrinsic fluorophores, such as nicotinamide adenine dinucleotide coenzyme (NADH), flavin adenine dinucleotide (FAD), nicotinamide adenine dinucleotide, collagen, elastin, and porphyrins can emit TPEF signals without labeling when biological tissues are exposed to near-infrared light. However, collagen, myosin, microtubules, starch, and cellulose in biological tissues have non-centrosymmetric structures that are prone to generating SHG signals. With the detection of strong TPEF and SHG signals from these intrinsic fluorescence chromophores, multi-channel cellular-level images can be created in unstained and unprocessed tissues; thus, MPM is the most promising candidate for development into a nondestructive tomography technique for clinical medicine.

In this study, we demonstrate the ability of MPM to identify meningiomas in the dura mater. First, the architectural and spectral features of normal and meningioma-infiltrated dura maters are characterized [Fig. 1(b)]. Second, the extent of dura mater changes after meningioma infiltration is quantitatively investigated through analysis of the content, orientation, and alignment of collagen [Fig. 1(c)]. Finally, using a self-developed image-processing algorithm, the precise borders of meningiomas in the dura mater are rapidly delineated, offering neurosurgeons more precise intraoperative resection guidance for meningiomas [Fig. 1(d)].

## 2 Materials and Methods

### 2.1 Sample Preparation

Seven fresh human dura mater samples and 10 meningioma-infiltrated dura mater samples from 10 patients were obtained from the Department of Neurosurgery at the First Affiliated Hospital of Fujian Medical University. All patients signed an informed consent form before the study. The Fujian Medical University Clinical Research Screening Committee for Studies Involving Human Subjects approved this study. After surgical removal by neurosurgeons, the samples were promptly delivered to the pathology laboratory within 30 min. They were processed into five serial slices using a cryostat microtome. The middle slice, ∼5  μm thick, was stained with hematoxylin and eosin (H&E) for histological imaging. The remaining four slices, each with ∼20  μm thickness, were placed between a coverslip and a microscope slide for multiphoton imaging. The pathological diagnostic results for the meningioma-infiltrated dura mater specimens and normal dura tissues were independently confirmed by two professional neuropathologists. Both neuropathologists utilized H&E images for pathology diagnosis and TPEF/SHG images for blind testing. The flow of the sample processing is shown in Fig. 1(a).

### 2.2 Multiphoton Microscopy Imaging System

The multiphoton imaging system used in this study has been described previously.^20^^,^^21^ Briefly, the system comprises a Zeiss LSM 510 microscope (Jena, Germany) and a Ti:sapphire femtosecond laser (Mira 900-F; Coherent, Inc.) tuned from 700 to 980 nm (Chameleon Ultra, Coherent, Inc., Santa Clara, California). In addition, a 63× Plan-Apochromat oil immersion objective (NA = 1.4; Zeiss, Jena, Germany) was used to focus the excitation beam onto the sample and collect the backward TPEF and SHG signals.

The system has two modes: lambda and channel. Both modes use the same detector, namely the META detector. This detector constitutes an optimized 32-channel photomultiplier tube array detector, covering a spectral width of ∼340  nm in the range of 377 to 716 nm. In the multi-channel mode, eight independent channels are included, and each channel can be optionally set for the detection of emission signals. Two channels were selected for the experiment. One channel covered the wavelength range of 389 to 419 nm for collecting SHG signals (color-coded green), and the other channel covered the range of 430 to 716 nm for collecting TPEF signals (color-coded red). The excitation wavelength was set at 810 nm, and the power ranged from 5 to 10 mW. All images were obtained at 2.56  μs/px, with the depth of 12-bit px. The detector in lambda mode can concurrently capture the spectrally resolved image and the corresponding spectra of 377 to 716 nm through emission lambda stacks. The flow of the sample processing is shown in Fig. 1(b).

### 2.3 Quantification of Collagen

**Fig. 2:**
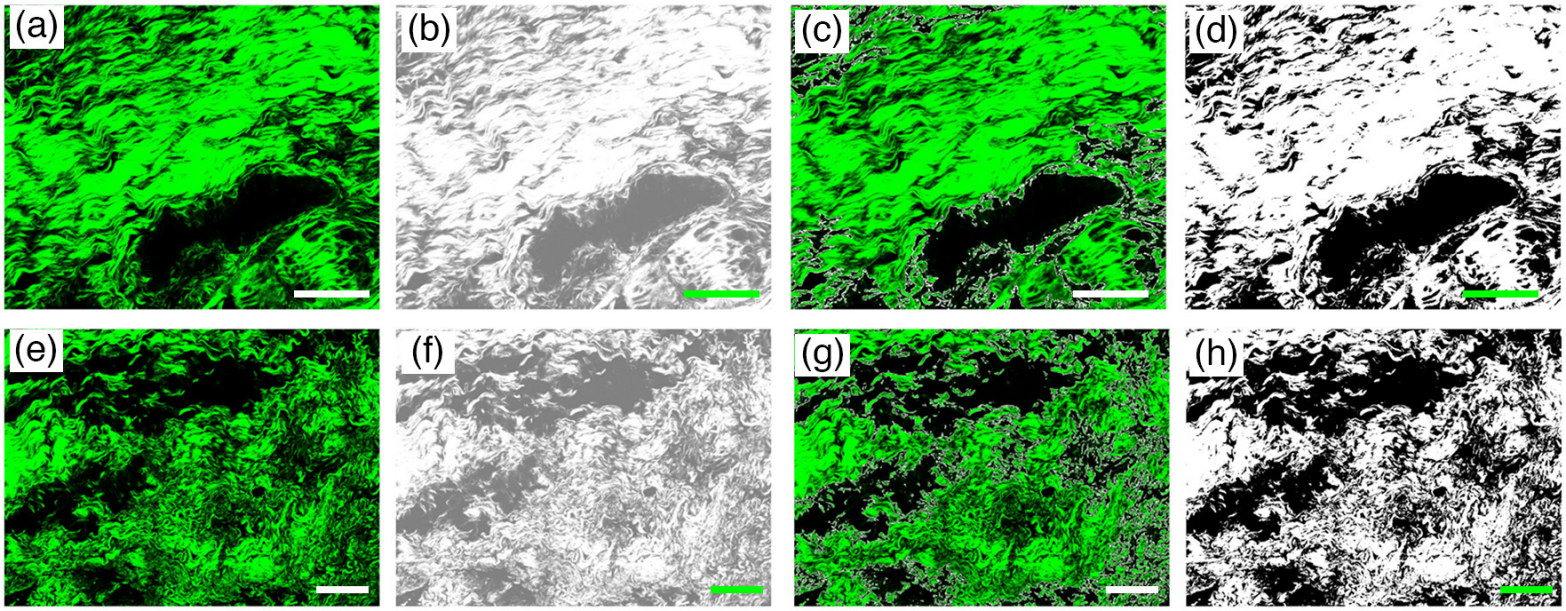
Quantification analysis of collagen content in normal dura mater and meningioma-infiltrated dura mater. (a)–(d) Original magnified SHG image, image enhancement result, segmentation result of collagen position, and final segmentation result of collagen content in normal dura mater. (e)–(h) Original magnified SHG image, image enhancement result, segmentation result of collagen position, and final segmentation result of collagen content in the meningioma-infiltrated dura mater. Scale bar: 100  μm.

In this study, SHG images were segmented and analyzed using MATLAB to automatically calculate collagen content (i.e., ratio of SHG to all pixels in each overlaid TPEF/SHG image). SHG images were realized as follows: first, the original SHG image was enhanced through Gaussian homogeneous filtering. The enhanced results were obtained using mathematical morphological processing and Otsu threshold segmentation. Finally, the segmentation template was employed to determine the collagen ratio based on the area occupied by the collagen. Figures 2(a)–2(h) present the typical collagen content segmentation results for the normal and meningioma-infiltrated dura mater. The program was used to identify the position of collagen and automatically calculate collagen density. At the cellular level, the images of collagen enhancement [Figs. 2(b) and 2(f)], collagen position segmentation [Figs. 2(c) and 2(g)], and final segmentation [Figs. 2(d) and 2(h)] are in good agreement with the original SHG images [Figs. 2(a) and 2(e)].

By contrast, the collagen fiber orientation of the dura mater tissue was investigated using a weighted orientation vector summation algorithm to quantitatively describe the orientation differences in collagen fibers, as previously detailed.^22^ Accordingly, the orientation distribution of collagen fibers in the normal and meningioma-infiltrated dura mater was measured and compared.

**Fig. 3:**
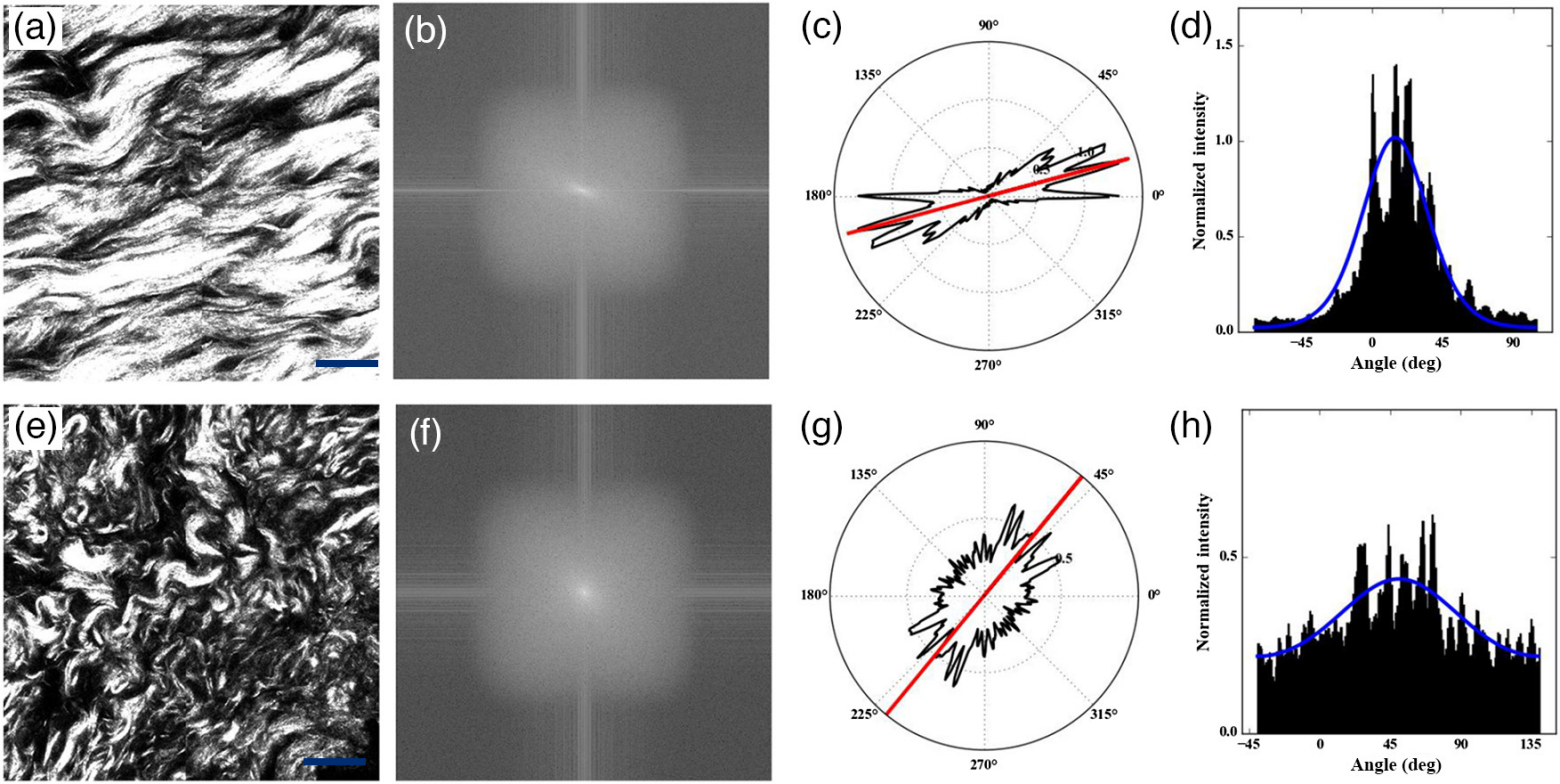
Representative collagen alignment results for normal dura mater and meningioma-infiltrated dura mater: (a), (e) original SHG images; (b), (f) fast Fourier transform power spectra; (c), (g) polar plots of collagen fiber orientation distributions (red line: mean orientation of fibers); (d), (h) collagen fiber alignments fitting semicircular von Mises distributions with parameter k. Scale bar: 20  μm.

Furthermore, fast Fourier transform and semicircular von Mises distribution computed using FiberFit (version:2.0) were performed to analyze the collagen fiber alignment.^23^ FiberFit is an open-source software package that provides the degree of fiber alignment by the parameter k, in which larger values of k indicate more aligned collagen fibers. Before utilizing FiberFit, the original SHG images were processed as follows. First, the images were sharpened, filtered with a Gaussian 3×3 smoothing operator, smoothed, and normalized. Each preprocessed image was converted to an 8-bit grayscale image. Finally, a despeckle operation was employed to remove noise from the converted images. Subsequently, the preprocessed images were imported into FiberFit. Figures 3(a)–3(h) show the representative collagen alignment quantification results for these two tissue types. The power spectra of the original SHG images were obtained to highlight the changes in pixel intensity [Figs. 3(b) and 3(f)]. The orientation of collagen fibers was determined using the radial sum [Figs. 3(c) and 3(g)). The alignment of collagen fibers was subsequently calculated by fitting semicircular von Mises distributions to the data [Figs. 3(d) and 3(h)]. The resulting distributions were parameterized using k.

### 2.4 Delineation of Tumor Boundaries

Visualization of the meningioma boundary is crucial during surgery. A precise boundary is highly beneficial for neurosurgeons to achieve complete lesion removal and reduce the risk of tumor recurrence. The obvious boundary of the meningioma in the dura mater is depicted as follows: first, the original overlaid TPEF/SHG image was changed to CIE Lab color space to obtain the luminance components. Second, the luminance component was converted to a grayscale image, and a Gaussian filter was used to remove impure and noisy areas. Third, fuzzy c-means clustering segmentation was adopted to obtain a rough boundary. Fourth, graph-cut segmentation was applied to obtain a more precise boundary. Finally, the boundary between the tumor and dura mater was marked by a white line.

### 2.5 Statistical Analysis

Different computed ratios were compared statistically using Student’s t-test in SPSS to obtain a P-value with a criterion of significance at P<0.5.

Blind analysis was performed by defining the sensitivity ($\text{sensitivity}=\frac{\mathrm{TP}}{\mathrm{TP}+\mathrm{FN}}$) and specificity ($\text{specificity}=\frac{\mathrm{TN}}{\mathrm{TN}+\mathrm{FP}}$) of the discrimination criteria. The variables were TP = true positive, FP = false positive, TN = true negative, and FN = false negative: TP = tumor tissue classified as tumor; FP = normal tissue classified as tumor; TN = normal tissue classified as normal; and FN = tumor tissue classified as normal.

## 3 Results

### 3.1 MPM Imaging and Multiphoton Spectral Analysis

**Fig. 4:**
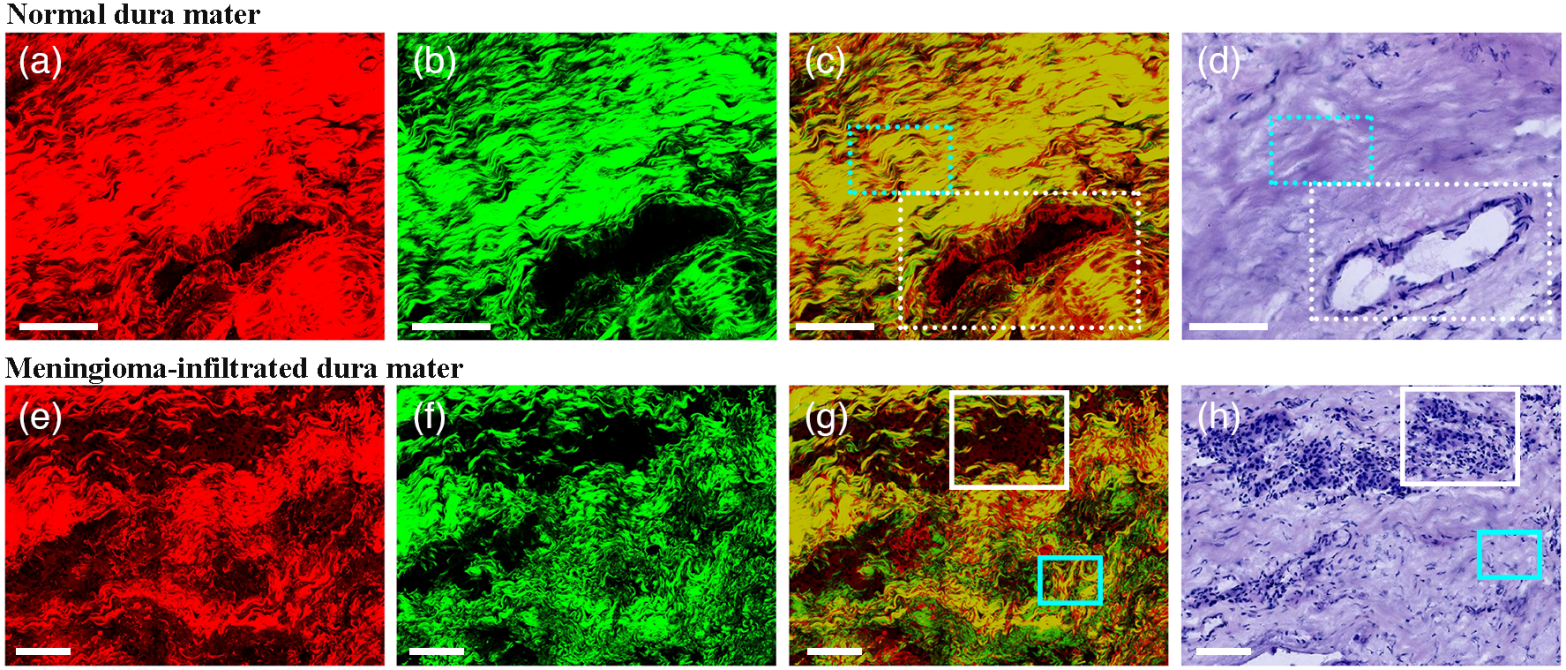
Representative TPEF image (color-coded red), SHG image (color-coded green), overlaid SHG/TPEF image, and the corresponding H&E-stained images of the normal and meningioma-infiltrated dura mater. (a)–(d) TPEF image, SHG image, overlaid SHG/TPEF image, and corresponding H&E-stained image of normal dura mater; (e)–(h) TPEF image, SHG image, the overlaid SHG/TPEF image, and the corresponding H&E-stained image of meningioma-infiltrated dura mater. Scale bar: 100  μm.

**Fig. 5:**
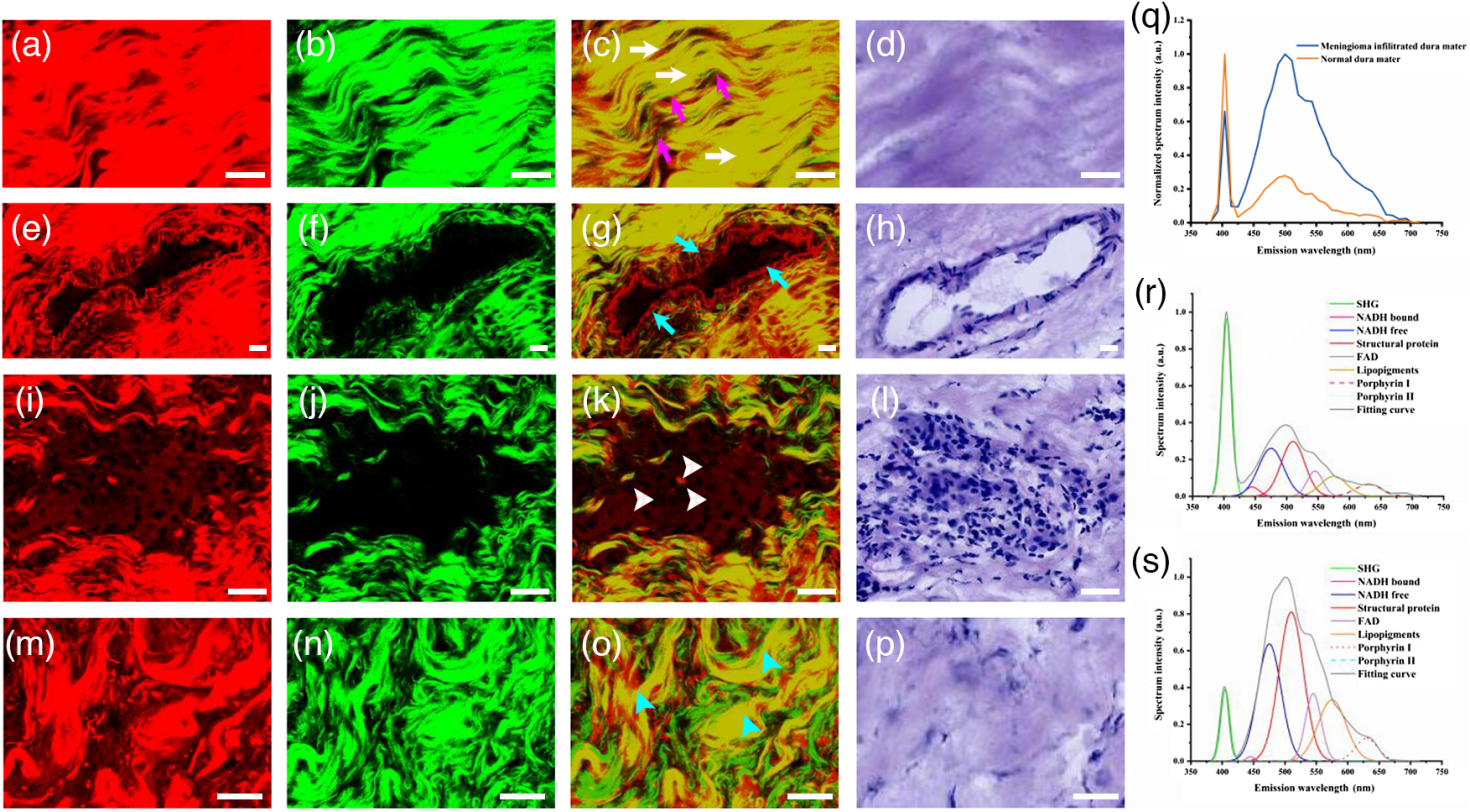
Microstructural details and multiphoton spectra of normal and meningioma-infiltrated dura mater. (a)–(d) Magnified TPEF image, SHG image, overlaid TPEF/SHG image, and corresponding H&E-stained image of the chosen area (cyan-dotted box) in Fig. 4(c). (e)–(h) Magnified TPEF image, SHG image, overlaid TPEF/SHG image, and corresponding H&E-stained image of the chosen area (white-dotted box) in Fig. 4(c). (i)–(l) Magnified TPEF, SHG, and overlaid TPEF/SHG image and the corresponding H&E-stained image of the chosen area (white box) in Fig. 4(g). (m)–(p) Magnified TPEF image, SHG image, overlaid TPEF/SHG image, and the corresponding H&E-stained image of the chosen area (cyan box) in Fig. 4(g). (q) Normalized multiphoton emission spectrum of normal dura mater and meningioma-infiltrated dura mater. Fitting spectral emission of (r) normal dura mater and (s) meningioma-infiltrated dura mater. White arrows: collagen fibers emitting comparable SHG and TPEF signals; purple arrows: collagen fibers only emitting SHG signals; cyan arrows: blood vessel; white arrowheads: tumor cells; cyan arrowheads: collagen bundles. Scale bar: 20  μm.

Figures 4(a)–4(d) display the typical TPEF, SHG, overlaid image, and corresponding H&E-stained image of normal dura mater. Figures 5(a)–5(h) show magnified images of the chosen area (cyan and white dotted boxes) in Fig. 4(c). The dura mater is characterized by abundant collagen fibers.^24^ These collagen fibers are arranged parallel to each other and condense into a compact collagen matrix. Figures 5(a)–5(d) clearly depict the microstructural details of the collagen fibers. Most collagen samples emitted comparable TPEF and SHG signals. A yellow color appeared when the SHG and TPEF signals were overlaid (white arrows). However, only a few collagen fibers (purple arrows) produced SHG signals (marked in green), suggesting that they might have different photochemical compositions. In Figs. 5(e)–5(h), the details of blood vessels in the normal dura mater are apparent. Blood vessels (cyan arrows) are easily recognizable because of the tube structure defined by elastin and collagen fibers. Collagen fibers exhibited strong SHG signals in blood vessel walls, whereas elastin fibers exhibited strong TPEF signals. This detailed tissue architectural information was in agreement with the corresponding H&E-stained images.

Figures 4(e)–4(h) display the typical TPEF, SHG, overlaid image, and corresponding H&E-stained images of the meningioma-infiltrated dura mater, respectively. Figures 5(i)–5(p) show magnified images of the chosen areas (white and cyan boxes) in Fig. 4(g). It can be seen that, in the large-area image, many tumor cells (white arrowheads) infiltrated and replaced the original collagen space, leading to a significant decrease in collagen content. Tumor-invading cells are depicted in Figs. 5(i)–5(l). Tumor cells can be identified by non-fluorescent nuclei and TPEF signals emitted by NADH, structural proteins, and FAD in the cytoplasm. Nuclei vary in size, shape, and appearance, ranging from round to oval to spindle shaped. Figures 5(m)–5(p) show the details of the collagen fibers (cyan arrowheads). The parallel arrangement of collagen transforms into a random orientation composed of twisted bundles of collagen. The tissue architecture details are consistent with the corresponding H&E-stained images. However, H&E-stained images, such as SHG images, cannot accurately characterize collagen changes like SHG images. To evaluate the capability of direct diagnosis based on TPEF/SHG images, TPEF/SHG images were provided to two senior neuropathologists for blind diagnoses. The senior pathologists achieved the specificity of 85.7% and sensitivity of 90.0% for discriminating tissues.

The above results indicate that the channel mode of MPM (TPEF imaging and SHG imaging) can be applied for morphological differentiation of meningioma-infiltrated dura mater from normal dura mater. To examine whether MPM has the potential to spectrally distinguish meningioma-infiltrated dura mater from the normal dura mater, the lambda mode of MPM was employed to obtain spectral images of the meningioma-infiltrated and normal dura mater. Figure 5(q) shows the normalized mean emission spectra of normal (yellow line) and meningioma-infiltrated dura mater (blue line) samples. Although they have specific spectral shapes, the spectra of both normal and meningioma-infiltrated dura mater exhibit two main peaks at 405 and 510 nm and six secondary peaks at 445, 475, 540, 570, 630, and 690 nm.^25^^,^^26^ Based on previous studies, the fluorescence peaks at 405 and 510 nm are attributed to the SHG signal and structural proteins. By contrast, the secondary peaks are related to protein-bound NADH, NADH-free, FAD, lipopigments, porphyrin I, and porphyrin II.^27^^–^^31^

To further the spectral analysis, a program was developed in MATLAB to fit the different endogenous molecules that emit fluorescence in dura mater. Figures 5(r) and 5(s) present the fitted data from the normal and meningioma-infiltrated groups, respectively. In the normal group, the relative SHG intensity is the highest, with the value of 0.973, followed by structural protein, NADH-free, and FAD, with values of 0.297, 0.260, and 0.137, respectively. By contrast, in the meningioma-infiltrated group, the fluorescence intensity of the structural protein was the highest with a value of 0.810, followed by NADH-free, SHG, and FAD with values of 0.637, 0.390, and 0.367, respectively. The obtained fitted spectra reveal that some endogenous molecule (SHG, NADH free, FAD) emission signals undergo a remarkable variation; therefore, we decided to track these molecular changes and focus our discrimination study on them to extract more parameters based on fitted emission signals.

Here, several molecular ratios related to the excited molecules were compared. Figure 4(a) shows the molecular ratios extracted from the normalized fitted spectra. Two molecular ratios for normal and meningioma-infiltrated cells were examined (Table 1): the SN ratio (SHG/[NADH free + protein-bound NADH]) and the FB ratio (NADH free/protein-bound NADH). As expected, there was a decrease in the SN ratio from the normal to meningioma-infiltrated dura mater, indicating a loss of collagen in the meningioma-infiltrated samples. Conversely, there was an increase in the FB ratio from the normal to meningioma-infiltrated dura mater. The SN and FB ratios showed significant differences between normal and meningioma-infiltrated dura maters. These differences in the emission spectra demonstrate the potential of MPM to qualitatively differentiate meningiomas infiltrating the normal dura mater. To enhance diagnosis, the two indicators, i.e., the SN ratio and FB ratio, were combined with the TPEF/SHG images. The blind diagnosis results achieved the specificity of 92.8% and sensitivity of 95.0% in discriminating tissues.

**Table 1:** Quantitative characterization from emission spectra.

Tissue	SN ratio			BF ratio		
	Mean	SD	P	Mean	SD	P
Normal dura mater	3.106	0.773	P<0.001	5.120	0.843	P<0.001
Dura mater with meningioma infiltration	0.590	0.493		25.828	8.117	

### 3.2 Quantification Analysis of Dura Mater Changes After Meningioma Infiltration

To further characterize the extent of changes in the dura mater after meningioma infiltration, the collagen content, orientation, and alignment were analyzed. The contrast in collagen content between the normal and meningioma-infiltrated dura mater is detailed in Table 2. Quantification analysis results indicate that the collagen content in the normal dura mater is far higher than that in the meningioma-infiltrated dura mater. Specifically, the collagen in the normal dura mater and meningioma-infiltrated dura mater is (0.853±0.073) and (0.553±0.133), respectively. The Student’s t-test was performed to determine significant differences between the collagen content in the normal dura mater and meningioma-infiltrated dura mater. Statistical analysis indicated that the difference between the two groups was statistically significant (P<0.001).

**Table 2:** Quantitative characterization from SHG images.

Tissue	Collagen content			k value		
	Mean	SD	P	Mean	SD	P
Normal dura mater	0.853	0.073	P<0.001	1.95	0.34	P<0.001
Dura mater with meningioma infiltration	0.553	0.133		0.35	0.11	

**Fig. 6:**
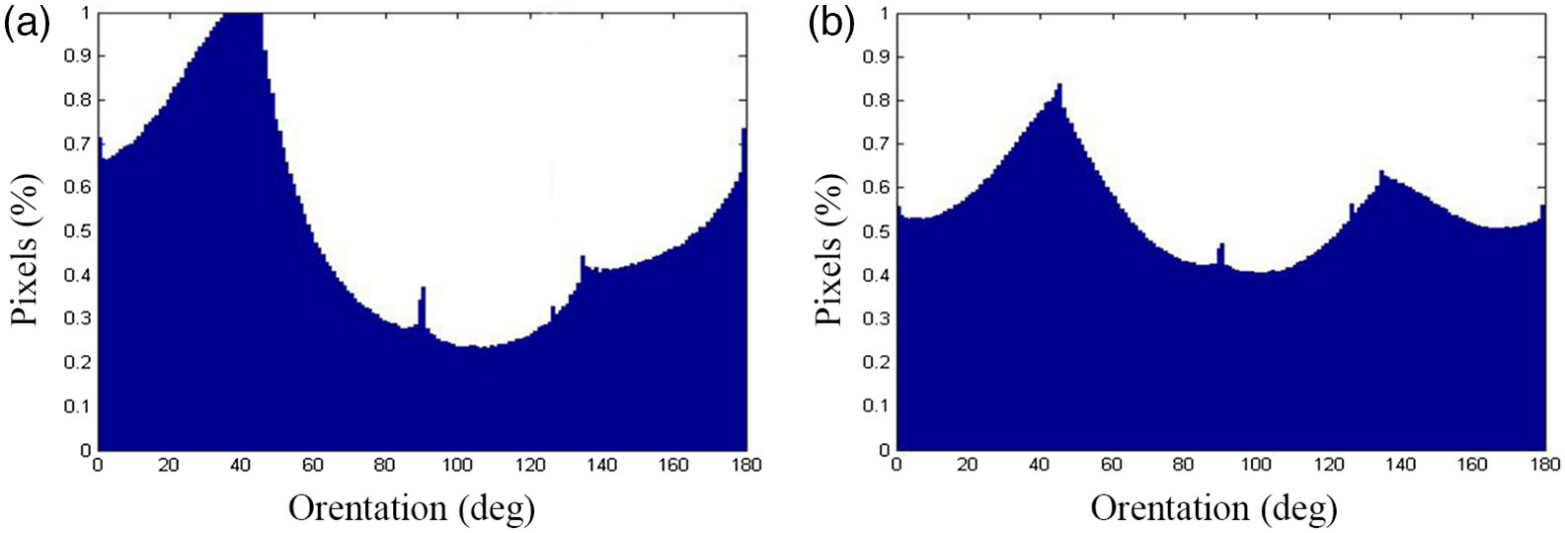
Collagen orientation distribution in (a) normal dura mater and (b) meningioma-infiltrated dura mater.

The results obtained using the weighted orientation vector summation algorithm are presented in Figs. 6(a) and 6(b). In the normal group, the orientation distribution of collagen fibers exhibits a single main peak, indicating that most collagen fibers were preferentially aligned in a particular orientation. The difference between the maximum and minimum values of the orientation distribution is 0.76, indicating a significant alignment of the collagen fibers in the chosen orientation. By contrast, the orientation distribution of the collagen fibers in the meningioma-infiltrated dura mater shows two main peaks, suggesting that the collagen fibers were aligned in two distinct orientations. The maximum and minimum values of the orientation distribution are only 0.42, corresponding to a more random fiber arrangement. These findings indicate that collagen fibers in the normal dura mater are more aligned than those in the meningioma-infiltrated dura mater. Moreover, these results highlight that the number of peaks and the difference between the maximum and minimum values of the orientation distribution can serve as quantitative indicators for distinguishing between normal and meningioma-infiltrated dura mater.

We used FiberFit to quantify the alignment of collagen fibers in the dura mater. The resulting distributions were parameterized using k. Table 2 shows the mean k values in the normal dura mater and the meningioma-infiltrated dura mater. The Student’s t-test in SPSS was used to determine significant differences among the k values. As shown in Table 2, the difference in k values between the two groups was significant (P<0.001), indicating a gradual decline in the meningioma-infiltrated dura mater. This implies that, when tumor cells infiltrate the adjacent dura mater, the collagen fibers undergo a transformation from a parallel arrangement to a random orientation. Thus, k is also a quantitative feature indicator for different normal and meningioma-infiltrated dura maters.

Together, the quantitative data confirmed that collagen was damaged and disordered as the meningioma invaded the surrounding dura mater. Data analysis revealed that collagen content, orientation distribution, and collagen fiber alignment can provide corresponding assessments of morphological changes in collagen in the invasive area of the dura mater. Therefore, collagen content, the number of peaks, the difference between the maximum and minimum values of the orientation distribution, and the k value in collagen fiber alignment can serve as diagnostic indicators to quantitatively detect meningiomas in the dura mater. To improve the diagnosis, regarding collagen content, number of peaks, and difference between the maximum and minimum values of the orientation distribution, the k value in the collagen fiber alignment was further incorporated into the TPEF/SHG images and spectral indicators (SN and FB ratios). The blind diagnosis results achieved the specificity of 100% and sensitivity of 100% in discriminating tissues.

### 3.3 Automated Identification of Tumor Boundary

**Fig. 7:**
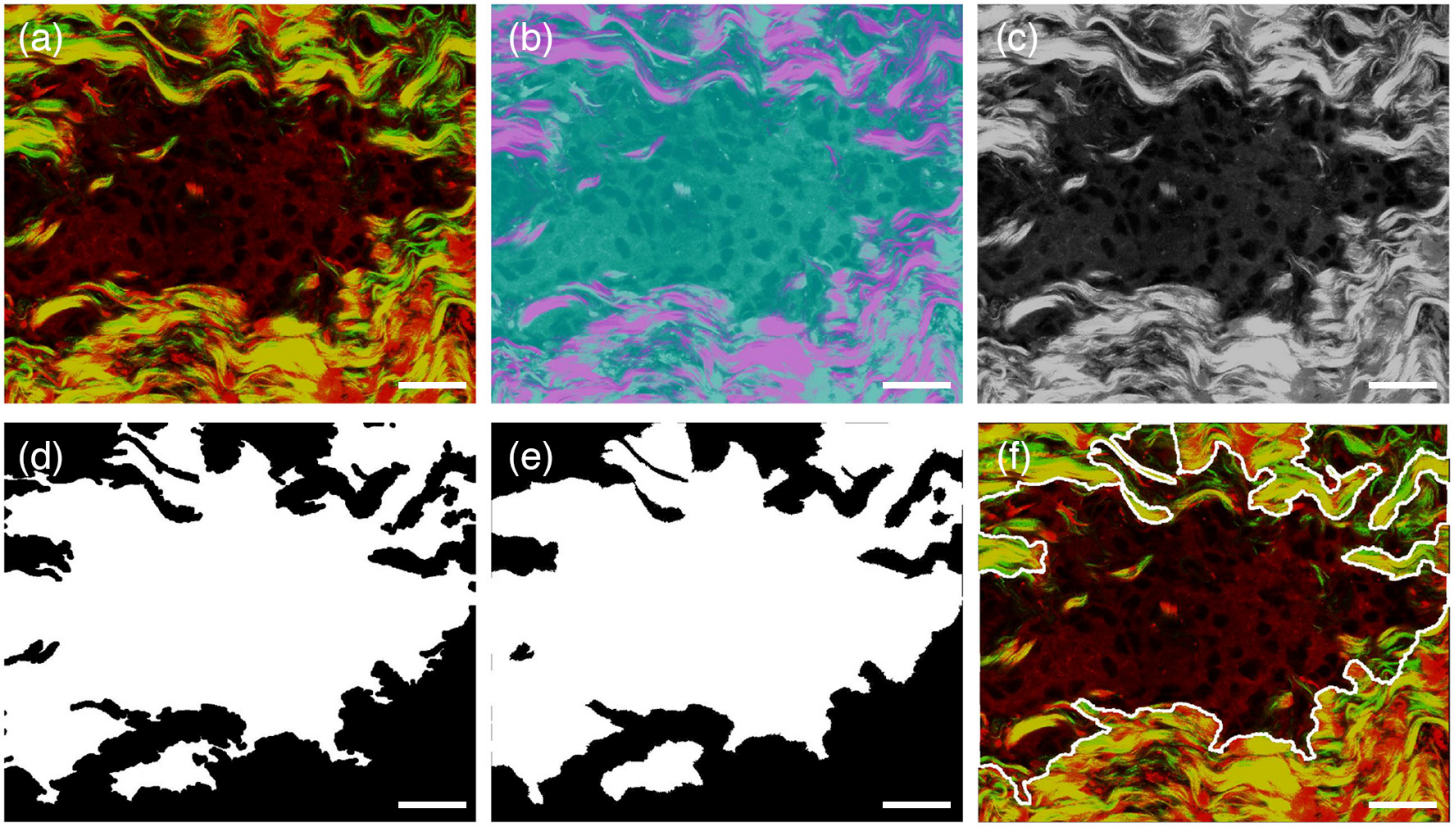
Identification of boundary of meningioma in dura mater. (a) Representative overlaid SHG/TPEF image; (b) result after converting to CIE Lab space; (c) result after converting to gray image and Gaussian filtering process; (d) rough boundary; (e) precise boundary; (f) final segment result. White line: the boundary automatically delineated by our proposed algorithm. Scale bar: 20  μm.

Another program was developed to automatically delineate the tumor boundary in the dura mater by overlaying TPEF/SHG images. The segmentation results for the boundary between the meningioma and the surrounding dura mater are displayed in Fig. 7. In the original overlaid TPEF/SHG image [Fig. 7(a)], the boundary between the meningioma and the surrounding dura mater is unclear. The results after conversion to the CIE Lab space are presented in Fig. 7(b). By converting to a gray image and applying a Gaussian filtering process, the noise is depressed, and the contrast between the meningioma and dura mater is enhanced, as shown in Fig. 7(c). After fuzzy C-means clustering segmentation, the tumor was automatically located and a rough boundary was obtained [Fig. 7(d)]. After graph-cut segmentation, a precise boundary was achieved, as exhibited in Fig. 7(e). As shown in Fig. 7(f), the proposed algorithm could correctly delineate the boundary from the complex dura mater background. As shown, the final segmentation result and original overlaid TPEF/SHG image [Fig. 7(a)] correct well at the cellular level. The research findings demonstrate that MPM combined with automated image analysis can distinguish meningioma from the dura mater and accurately delineate the boundary of the tumor.

## 4 Discussion

In this study, MPM was applied for label-free imaging of the dura mater. The morphological features of normal dura mater and meningioma-infiltrated dura mater were first investigated using the multi-channel mode. Our current study indicates that FAD, NADH, and structural proteins in meningioma cells can produce strong TPEF signals, whereas collagen fibers in the dura mater can emit strong SHG signals. Through analyzing the SHG and TPEF signals from intrinsic fluorescent molecules, MPM can detect meningioma cells in the dura mater as well as reveal morphological details and alterations, including reduction in collagen content, changes in collagen orientation, and invasion of meningioma cells. In addition, the proposed method has the potential to quantify the differences between normal and meningioma-infiltrated dura mater by calculating the collagen content, orientation, and alignment, thereby providing a more objective basis for pathologists and neurosurgeons.

The spectroscopic features of normal and meningioma-infiltrated dura maters were also investigated using multiphoton spectral analysis. Significant differences were observed between the normalized emission spectra of the normal and meningioma-infiltrated dura mater, indicating remarkable variations in the molecular composition of the two tissues. Compared with the meningioma-infiltrated dura mater, the multiphoton spectra of the normal dura mater exhibited a high SHG signal intensity at 405 nm. By contrast, the levels of free NADH, structural proteins, and FAD in meningioma-infiltrated dura mater tissues were significantly higher than those in normal dura mater tissues.

The results of the molecular variation were revealed through normalized emission spectra between the normal and meningioma-infiltrated dura mater tissues. SHG and seven fluorophores, including protein-bound NADH, NADH-free, structural protein, FAD, lipopigments, porphyrin I, and porphyrin II, were chosen and fitted by Gaussian curves based on a literature review.^28^ According to the fitted emission spectra, two molecular ratios related to the excited molecules were compared: the SN and BF ratios.

The SN ratio decreased from normal to meningioma-infiltrated dura mater, indicating a decrease in collagen content and an increase in NADH content in meningioma-infiltrated dura mater samples. Collagen is the primary component of the dura mater. In normal dura mater, collagen fibers and bundles are densely packed and neatly arranged, contributing to greater tensile strength and stiffness. However, in the meningioma-infiltrated dura mater, the original tightly interwoven arrangement is replaced by tumor cells, leading to a reduction in collagen content. In addition, the collagen crosslinks, which determine the TPEF signal of collagen, were dramatically reduced, explaining the dominant SHG signal and weaker TPEF signal of collagen in the meningioma-infiltrated dura mater [Fig. 4(g)], compared with the SHG signal and TPEF signal in the normal case [Fig. 4(c)].

In the meningioma-infiltrated dura mater, a clear increase in NADH content was observed. NADH is a coenzyme involved in cellular respiration and energy production and is essential for cell function. Monitoring NADH content in tissues provides crucial information about the metabolic state. Previous reports have shown increased NADH content in tumors.^32^^,^^33^ Our previous studies on normal and glioma human brain tissues also demonstrated higher NADH levels in gliomas.^34^ In this study, when the dura mater was invaded by tumor cells, the metabolic activity increased, which explains the higher NADH content in the meningioma-infiltrated dura mater.

The FB ratio is also an indicator of cellular metabolism, with higher values indicating a shift toward glycolysis and greater NADH content, reflecting higher metabolism. In our study, the BF ratio in normal dura mater was measured at 5.120±0.843, whereas in meningioma-infiltrated dura mater, it increased to 25.828±8.117. This increase was attributed to a significant increase in the absolute amount of free NADH and NADH, as the absolute amount of protein-bound NADH remained relatively stable. ^31^ These significant differences in fluorescence intensity can be considered potential standards for the detection of meningioma-infiltrated dura mater. Moreover, the variations observed in the emission spectra served as quantitative indicators to differentiate between normal and meningioma-infiltrated dura mater tissues. CN, FB, and redox ratios can also be used as quantitative indicators to differentiate between normal and meningioma-infiltrated dura mater tissues.

Further, the MPM was combined with a custom-developed automated image processing program in this study to automatically locate the tumor in the dura mater and provide a rapid method for accurately delineating the tumor boundary. MPM is highly sensitive to blood vessels, as elastin in the vessel wall can generate TPEF signals and collagen can emit SHG signals, which allows for label-free visualization. In this context, our proposed technique could assist neurosurgeons in completely, thoroughly, and rapidly excising lesions while minimizing major bleeding and preserving as much surrounding normal tissue as possible, ultimately leading to faster recovery with a lower risk of infection. Hence, our proposed method not only provides a histopathological examination of meningiomas in the dura mater but also contributes to the automatic and precise resection of meningiomas.

Recent advancements in MPM in fundamental clinical research have facilitated the evolution of multiphotonic systems toward multimodality^35^ and miniaturization,^36^^,^^37^ presenting a range of advanced imaging techniques with increased endogenous signal contrast, faster scanning speeds, and smaller device sizes. Our findings provide a theoretical basis for future clinical MPM surgeries for meningioma. The adoption of combined MPM and image-processing algorithms will promote the development of MPM diagnostic systems. With the optimization of the GRIN lens,^38^ photonic crystal fiber,^39^ laser source,^19^ intelligent image algorithms,^40^ and intelligent quantitative miniaturized multiphoton endoscopy systems, neurosurgeons can accurately diagnose meningioma lesions in real time and perform their complete excision of meningioma lesions in the operating room.

## References

[1] Fathi A.-R. Roelcke U., “Meningioma,” Curr. Neurol. Neurosci. 13(4), 337 (2013). 10.1007/s11910-013-0337-4 23463172

[2] Jääskeläinen J., “Seemingly complete removal of histologically benign intracranial meningioma: late recurrence rate and factors predicting recurrence in 657 patients. A multivariate analysis,” Surg. Neurol. 26(5), 461–469 (1986). SGNRAI 0090-3019 10.1016/0090-3019(86)90259-4 3764651

[3] Mirimanoff R. O. et al., “Meningioma: analysis of recurrence and progression following neurosurgical resection,” J. Neurosurg. 62(1), 18–24 (1985). JONSAC 0022-3085 10.3171/jns.1985.62.1.0018 3964853

[4] Mahmood A. Qureshi N. H. Malik G. M., “Intracranial meningiomas: analysis of recurrence after surgical treatment,” Acta Neurochir. 126(2–4), 53–58 (1994). 10.1007/BF01476410 8042555

[5] Simpson D., “The recurrence of intracranial meningiomas after surgical treatment,” J. Neurol. Neurosurg. Psychiatry 20(1), 22–39 (1957). JNNPAU 0022-3050 10.1136/jnnp.20.1.22 13406590 PMC497230

[6] Borovich B. Doron Y., “Recurrence of intracranial meningiomas: the role played by regional multicentricity,” J. Neurosurg. 64(1), 58–63 (1986). JONSAC 0022-3085 10.3171/jns.1986.64.1.0058 3941351

[7] Watts J. et al., “Magnetic resonance imaging of meningiomas: a pictorial review,” Insights Imaging 5(1), 113–122 (2014). 10.1007/s13244-013-0302-4 24399610 PMC3948902

[r8] Lin B.-J. et al., “Correlation between magnetic resonance imaging grading and pathological grading in meningioma,” J. Neurosurg. 121(5), 1201–1208 (2014). JONSAC 0022-3085 10.3171/2014.7.JNS132359 25148010

[9] Miyoshi K. et al., “Predicting the consistency of intracranial meningiomas using apparent diffusion coefficient maps derived from preoperative diffusion-weighted imaging,” J. Neurosurg. 135(3), 969–976 (2021). JONSAC 0022-3085 10.3171/2020.6.JNS20740 33186907

[10] Fereidouni F. et al., “Microscopy with ultraviolet surface excitation for rapid slide-free histology,” Nat. Biomed. Eng. 1(12), 957–966 (2017). 10.1038/s41551-017-0165-y 31015706 PMC6223324

[11] Liu J. T. C. et al., “Harnessing non-destructive 3D pathology,” Nat. Biomed. Eng. 5(3), 203–218 (2021). 10.1038/s41551-020-00681-x 33589781 PMC8118147

[12] Pahlevaninezhad H. et al., “Nano-optic endoscope for high-resolution optical coherence tomography *in vivo*,” Nat. Photonics 12(9), 540–547 (2018). NPAHBY 1749-4885 10.1038/s41566-018-0224-2 30713581 PMC6350822

[13] Wong T. T. W. et al., “Fast label-free multilayered histology-like imaging of human breast cancer by photoacoustic microscopy,” Sci. Adv. 3(5), e1602168 (2017). STAMCV 1468-6996 10.1126/sciadv.1602168 28560329 PMC5435415

[14] Li J. et al., “Resolution-enhanced intensity diffraction tomography in high numerical aperture label-free microscopy,” Photonics Res. 8(12), 1818 (2020). 10.1364/PRJ.403873

[15] Denk W. Strickler J. Webb W., “Two-photon laser scanning fluorescence microscopy,” Science 248(4951), 73–76 (1990). SCIEAS 0036-8075 10.1126/science.2321027 2321027

[r16] Larson A. M., “Multiphoton microscopy,” Nat. Photonics 5(1), 1–1 (2010). NPAHBY 1749-4885 10.1038/nphoton.an.2010.2

[r17] You S. et al., “Label-free deep profiling of the tumor microenvironment,” Cancer Res. 81(9), 2534–2544 (2021). CNREA8 0008-5472 10.1158/0008-5472.CAN-20-3124 33741692 PMC8137645

[r18] Chen D. et al., “Association of the collagen signature in the tumor microenvironment with lymph node metastasis in early gastric cancer,” JAMA Surg. 154(3), e185249 (2019). 10.1001/jamasurg.2018.5249 30698615 PMC6439641

[19] Lefort C., “A review of biomedical multiphoton microscopy and its laser sources,” J. Phys. D: Appl. Phys. 50(42), 423001 (2017). JPAPBE 0022-3727 10.1088/1361-6463/aa8050

[20] Zhuo S. et al., “Label-free monitoring of colonic cancer progression using multiphoton microscopy,” Biomed. Opt. Express 2(3), 615 (2011). BOEICL 2156-7085 10.1364/BOE.2.000615 21412466 PMC3047366

[21] Zhuo S. et al., “Multimode nonlinear optical imaging of the dermis in *ex vivo* human skin based on the combination of multichannel mode and Lambda mode,” Opt. Express 14(17), 7810 (2006). OPEXFF 1094-4087 10.1364/OE.14.007810 19529150

[22] Quinn K. P. Georgakoudi I., “Rapid quantification of pixel-wise fiber orientation data in micrographs,” J. Biomed. Opt. 18(4), 046003 (2013). JBOPFO 1083-3668 10.1117/1.JBO.18.4.046003 23552635 PMC3639785

[23] Morrill E. E. et al., “A validated software application to measure fiber organization in soft tissue,” Biomech. Model. Mechanobiol. 15(6), 1467–1478 (2016). BMMICD 1617-7959 10.1007/s10237-016-0776-3 26946162 PMC5328598

[24] Reina M. A. et al., “Ultrastructure of spinal dura mater,” in Atlas of Functional Anatomy for Regional Anesthesia and Pain Medicine: Human Structure, Ultrastructure and 3D Reconstruction Images, De Andrés J. A. et al., Eds., pp. 411–434, Springer International Publishing (2015).

[25] Ramanujam N., “Fluorescence spectroscopy of neoplastic and non-neoplastic tissues,” Neoplasia 2(1–2), 89–117 (2000). 10.1038/sj.neo.7900077 10933071 PMC1531869

[26] Monici M., “Cell and tissue autofluorescence research and diagnostic applications,” Biotechnol. Annu. Rev. 11, 227–256 (2005). 10.1016/s1387-2656(05)11007-2 16216779

[27] Zanello M. et al., “Multimodal optical analysis of meningioma and comparison with histopathology,” J. Biophotonics 10(2), 253–263 (2016). 10.1002/jbio.201500251 26871683

[r28] Mehidine H. et al., “Molecular changes tracking through multiscale fluorescence microscopy differentiate meningioma grades and non-tumoral brain tissues,” Sci. Rep. 11(1), 3816 (2021). 10.1038/s41598-020-78678-4 33589651 PMC7884789

[r29] Zanello M. et al., “Multimodal optical analysis discriminates freshly extracted human sample of gliomas, metastases and meningiomas from their appropriate controls,” Sci. Rep. 7(1), 41724 (2017). 10.1038/srep41724 28150726 PMC5288720

[r30] Poulon F. et al., “Multimodal analysis of central nervous system tumor tissue endogenous fluorescence with multiscale excitation,” Front. Phys. 6, 109 (2018). FRPHAY 0429-7725 10.3389/fphy.2018.00109

[31] Croce A. C. Bottiroli G., “Autofluorescence spectroscopy and imaging: a tool for biomedical research and diagnosis,” Eur. J. Histochem. 58(4), 2461 (2014). EJHIE2 0391-7258 25578980 10.4081/ejh.2014.2461 PMC4289852

[32] Schaefer P. M. et al., “NADH autofluorescence: a marker on its way to boost bioenergetic research,” Cytometry Part A 95(1), 34–46 (2018).1552-4922 10.1002/cyto.a.23597 30211978

[33] Skala M. C. et al., “*In vivo* multiphoton microscopy of NADH and FAD redox states, fluorescence lifetimes, and cellular morphology in precancerous epithelia,” Proc. Natl. Acad. Sci. U. S. A. 104(49), 19494–19499 (2007). 10.1073/pnas.0708425104 18042710 PMC2148317

[34] Fang N. et al., “Quantitative assessment of microenvironment characteristics and metabolic activity in glioma via multiphoton microscopy,” J. Biophotonics 12(10), e201900136 (2019). 10.1002/jbio.201900136 31251837

[35] You S. et al., “Intravital imaging by simultaneous label-free autofluorescence-multiharmonic microscopy,” Nat. Commun. 9(1), 2125 (2018). NCAOBW 2041-1723 10.1038/s41467-018-04470-8 29844371 PMC5974075

[36] Dilipkumar A. et al., “Label-free multiphoton endomicroscopy for minimally invasive *in vivo* imaging,” Adv. Sci. 6(8), 1801735 (2019). 10.1002/advs.201801735 PMC6468963 31016109

[37] Zong W. et al., “Publisher correction: miniature two-photon microscopy for enlarged field-of-view, multi-plane and long-term brain imaging,” Nat. Methods 18(2), 220–220 (2021).1548-7091 10.1038/s41592-021-01066-x 33479525

[38] Lukic A. et al., “Endoscopic fiber probe for nonlinear spectroscopic imaging,” Optica 4(5), 496–501 (2017). 10.1364/OPTICA.4.000496

[39] Tu H. et al., “Stain-free histopathology by programmable supercontinuum pulses,” Nat. Photonics 10(8), 534–540 (2016). NPAHBY 1749-4885 10.1038/nphoton.2016.94 27668009 PMC5031149

[40] Bi W. L. et al., “Artificial intelligence in cancer imaging: clinical challenges and applications,” CA: Cancer J. Clin. 69(2), 127–157 (2019). 10.3322/caac.21552 30720861 PMC6403009

